# Cancer Missense Mutations Alter Binding Properties of Proteins and Their Interaction Networks

**DOI:** 10.1371/journal.pone.0066273

**Published:** 2013-06-14

**Authors:** Hafumi Nishi, Manoj Tyagi, Shaolei Teng, Benjamin A. Shoemaker, Kosuke Hashimoto, Emil Alexov, Stefan Wuchty, Anna R. Panchenko

**Affiliations:** 1 National Center for Biotechnology Information, National Library of Medicine, National Institutes of Health, Bethesda, Maryland, United States of America; 2 Department of Genetics, Washington University School of Medicine, St. Louis, Missouri, United States of America; 3 Computational Biophysics and Bioinformatics, Department of Physics, Clemson University, Clemson, South Carolina, United States of America; 4 Omics Science Center, RIKEN, Yokohama, Japan; Koc University, Turkey

## Abstract

Many studies have shown that missense mutations might play an important role in carcinogenesis. However, the extent to which cancer mutations might affect biomolecular interactions remains unclear. Here, we map glioblastoma missense mutations on the human protein interactome, model the structures of affected protein complexes and decipher the effect of mutations on protein-protein, protein-nucleic acid and protein-ion binding interfaces. Although some missense mutations over-stabilize protein complexes, we found that the overall effect of mutations is destabilizing, mostly affecting the electrostatic component of binding energy. We also showed that mutations on interfaces resulted in more drastic changes of amino acid physico-chemical properties than mutations occurring outside the interfaces. Analysis of glioblastoma mutations on interfaces allowed us to stratify cancer-related interactions, identify potential driver genes, and propose two dozen additional cancer biomarkers, including those specific to functions of the nervous system. Such an analysis also offered insight into the molecular mechanism of the phenotypic outcomes of mutations, including effects on complex stability, activity, binding and turnover rate. As a result of mutated protein and gene network analysis, we observed that interactions of proteins with mutations mapped on interfaces had higher bottleneck properties compared to interactions with mutations elsewhere on the protein or unaffected interactions. Such observations suggest that genes with mutations directly affecting protein binding properties are preferably located in central network positions and may influence critical nodes and edges in signal transduction networks.

## Introduction

Most cancers are characterized by genomic instability which is considered to be one of the important factors driving tumor development [1]. These genetic perturbations potentially lead to abnormal oncogene activation and/or tumor suppressor gene inactivation. According to the concept of “oncogene addiction”, cancer cells depend on the activity of a single or a few oncogenes for their proliferation and survival [2]. Altered activity of oncogenes and tumor suppressors may be caused by gene amplifications, enhanced or decreased transcription or translation. At the same time, missense mutations might also play a very important role in carcinogenesis [3]. While contributing significantly to tumorigenesis, majority of mutations are considered neutral (*i.e.* “passenger” mutations), and only a few are under positive selection in cancer cells (*i.e.* “driver” mutations) [3], [4]. Various methods have been applied to predict the deleterious effects of mutations [5], [6], to find positively selected mutants and to distinguish driver from passenger mutations [7], [8]. However, their predictive power remains limited, largely depends on the level of evolutionary conservation [9] and the background mutation rate which is difficult to determine for each sample [10]. Moreover, recent results suggest that a large majority of single nucleotide variations predicted to be functionally important are rare (with minor allele frequency less than 0.5%) [11], making such rare disease-associated variants difficult to detect.

Many signaling networks are deregulated in cancer and involve a dense network of protein-protein interactions. Therefore, the characterization of cancer-related protein interaction networks is essential for our understanding of the molecular mechanisms of carcinogenesis. Recently, new strategies were proposed to identify key network modules and driver oncogenes by combining copy number variations, missense mutations and mapping potential oncogenic driver genes onto high-throughput protein-protein interaction networks [12], [13], [14]. As a result of these studies, novel cancer-related genes and functionally-related gene modules targeted by driver cancer mutations were identified [13], [14], [15].

Moreover, proteins recognize and bind their specific targets in a highly regular manner and the specificity of these interactions is largely determined by structural and physico–chemical properties of binding interfaces. Recently, structural complexes of disease- and cancer-related proteins were analyzed [16], [17], [18], [19], showing that disease-related protein complexes have distinct binding properties; in particular, they contain multiple interface patches, enabling interactions with many other proteins [16], and mutations on different patches might have caused pleiotropic disease effects [20]. In addition, many disease mutations are located on protein-protein interfaces [21], [22], [23], a tendency that is especially pronounced for cancer missense mutations [20]. Such observations generally emphasize the importance of studying the effects of cancer mutations on protein interactions and on their binding interfaces in particular.

Many oncogenes, tumor suppressors and their mutations have been identified as key players in cancer signaling events. However, only a few have been found in different types of cancer simultaneously. Such heterogeneity complicates the identification of key players that provide selective advantages to tumor cells. In our study we utilized a set of mutations derived from glioblastoma patients, allowing us to narrow down the heterogeneity of phenotypic response to better understand genotype-phenotype relationships. Glioblastoma is the most malignant form of brain tumors according to WHO classification [24]. Recently, The Cancer Genome Atlas (TCGA) and other projects provided mutation data of glioblastoma patients on a large scale [25], [26]. Eight potential driver genes were identified in glioblastomas, and mapping mutated genes on biochemical pathways indicated several prevalent pathways that contained mutated driver genes [25], [26], [27]. Specifically, genome alterations that were found in several key pathways were observed to be mutually exclusive to each pathway, pointing to the sufficient selective advantage of these few alterations for cancer cells [25].

**Figure 1:**
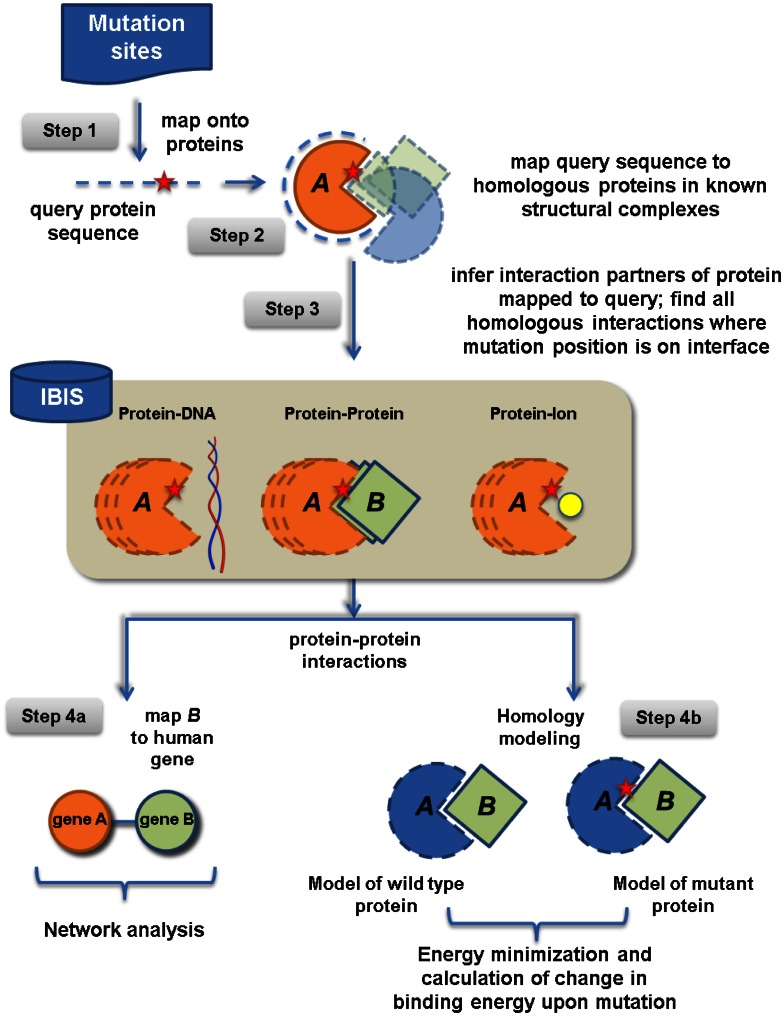
Mapping the human interactome and glioblastoma mutations on binding interfaces. In step 1 we mapped 695 missense mutations from 598 human genes to protein sequences. Subsequently, query protein sequences were aligned to homologous, experimentally determined structural complexes (step 2), allowing us to infer query-specific interactions with other proteins, nucleic acids and ions (step 3). For protein-protein interactions, we mapped interaction partners to their corresponding human proteins (step 4a), allowing us to find 160 protein interactions between 150 genes with mutations affecting their interaction interfaces. In step 4b, we compared the structures of the unperturbed wild-type protein and the mutated protein by performing energy minimization calculations and determining binding energy differences.

Recently, we mapped the human protein interactome using structural complexes which allowed us to decipher the effect of glioblastoma missense mutations on protein-protein, protein-nucleic acid, protein-ion binding interfaces and phosphorylation sites in this study (Figure 1). Here, we show that mutations on binding interfaces result in more drastic changes of amino acid physico-chemical properties than mutations that cannot be mapped on interfaces. Moreover, we found that mutations on protein-protein interfaces have overall destabilizing effects and mostly affect the electrostatic component of binding energy as well as the topology of protein-protein interaction networks. Importantly, we identify possible driver mutations and genes, some of which are specific to nervous system functioning. We complement our findings by suggesting the molecular mechanisms of the phenotypic effect of mutations.

## Results and Discussion

### Cancer Mutations might Affect Phosphorylation Sites

Many proteins that play an important role in cancer may also participate in signaling pathways, typically mediating signals through phosphorylation events. Previously, somatic cancer mutations were shown to potentially cause gain or loss of phosphorylation sites [29]. Therefore, we hypothesized that glioblastoma mutations may also affect phosphorylation sites, potentially disrupting the flow of signals through the loss of sites. We collected 2,825 phosphorylation sites from the PhosphoSitePlus [30], Phospho.ELM [31] and PHOSIDA [32] databases which were further verified by GPS software [33]. While 94 mutation sites in Ser/Thr/Tyr residues could be potentially phosphorylated, we found that 6 out of 94 sites significantly overlapped with phosphorylation sites (Fisher exact test p-value = 0.028, Table S1 in File S1). Indeed, phosphorylation may be accompanied by the changes in local site environment or global conformation, lead to protein activation or inactivation and modulate the strength of protein or DNA interactions [34]. Therefore, mutation of a phosphorylation site may result in the loss of these important functional properties, as exemplified by the loss of phosphorylation site Ser 313 in P53 that regulates binding to DNA.

### Effect of Glioblastoma Mutations on Protein Binding

**Figure 2:**
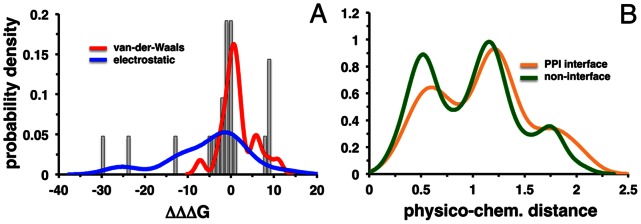
The effect of mutations on protein binding. (**A**) Distribution of binding energy difference upon mutation for electrostatic and van-der-Waals components. The electrostatic component of binding energy was significantly shifted toward negative values compared to the van-der-Waals component (p-value = 1.3×10^−3^) (**B**) Distributions of physico-chemical distances between amino acids that correspond to glioblastoma mutations on protein-protein interfaces and non-interface regions. Distributions that referred to amino acid substitutions on protein-protein interfaces had significantly larger distances compared to non-interface regions (p-value = 0.011).

We integrated mutated genes in a structurally inferred protein interaction network and estimated the effect of these mutations on such a network. Specifically, we constructed mutant structural models (see Methods) and calculated the differences of binding energies that were caused by the corresponding amino acid substitutions. We found a negative average binding energy difference of *ΔΔΔG* = −2.54 kcal/mol, pointing to an overall destabilizing effect of mutations on protein-protein complexes in glioblastomas (Figure 2A, Table 1). Furthermore, the electrostatic component of binding energy was shifted towards negative values compared to zero (p-value = 0.007) and compared to the van-der-Waals component (p-value = 0.0013). Meanwhile, the van-der-Waals component itself did not show an overall de- or over-stabilizing effect. While several applications have been developed to predict the effect of mutations on protein stability, we compared our results to FoldX, allowing us to observe a significant, although not very high, correlation between the *ΔΔΔG* values of both approaches (Figure S1B in File S1, Pearson’s r_P_ = 0.4÷0.77, p-value <0.01). These differences may arise from the fact that FoldX uses an empirical potential calibrated on the set of experimental changes of unfolding energy in the presence of mutations. Furthermore, FoldX is not explicitly trained on disease mutations and binding energy changes and does not account for the mutation induced conformational changes of the protein backbone.

**Table 1:** List of representative genes with mutation sites located on different types of protein binding interfaces.

Gene	Protein GI	Orig AA	Mut Pos	New AA	Structure ofhomolog	Structure ofbinding partner	Dist. Phys	dddG
**Protein-protein**								
**ABL2**	6382062	P	487	L	2RF9_A	2RF9_C	1.17	0.695
**EPHA2**	32967311	G	111	D	3MBW_A	3MBW_B	1.28	0.14
**IDH1**	28178825	R	132	H	1T09_A	1T09_B	1.03	8.64
**NLGN2**	30840978	E	577	K	3BIW_A	3BIW_D	1.54	−1.24
**NRAS**	4505451	G	12	D	1NVU_R	1NVU_S	1.28	−4.24
**RAB3C**	19923985	R	49	H	2P5S_B	2P5S_A	1.03	−1.48
**RAC2**	4506381	D	47	Y	2V55_B	2V55_A	1.40	−2.14
**RAD52**	109637798	R	46	K	1KN0_A	1KN0_K	0.57	9.04
**TP53**	120407068	P	177	S	1YCS_A	1YCS_B	1.19	−1.29
**TP53**	120407068	R	248	Q,W	1YCS_A	1YCS_B	1.14,1.75	−24.31, −29.69
**TP53**	120407068	R	273	H,C	1YCS_A	1YCS_B	1.03,1.78	−3.44, −13.45
**TP53**	120407068	D	281	A	1YCS_A	1YCS_B	1.16	9.03
**Protein-DNA**								
**BCL11A**	20336305	R	740	C	2DRP_A	DNA	1.78	−0.02
**PAX9**	7242167	R	26	W	6PAX_A	DNA	1.75	−0.05
**TP53**	120407068	R	248	Q,W	3KMD_A	DNA	1.14,1.75	0.36, 1.27
**TP53**	120407068	R	273	H,C	3KMD_A	DNA	1.03,1.78	−0.84, −0.54
**TP53**	120407068	A	276	V	3KMD_A	DNA	0.573	0.28
**ZIK1**	77736604	T	393	A	2I13_A	DNA	0.55	−0.40
**ZNF339**	40807463	T	222	M	1MEY_C	DNA	0.81	−0.11
**Protein-RNA**								
**ELAVL2**	115511032	G	167	D	1FXL_A	RNA	1.28	–
**KLK9**	29366812	Y	240	D	3DD2_H	RNA	1.40	–
**RBMS3**	51317353	I	166	V	1FXL_A	RNA	0.42	–
**RPL11**	15431290	R	75	X	3KCR_F	RNA	-	–
**Protein-ion**								
**ADAMTS17**	110611170	D	434	G	1KUG_A	Cd^2+^	1.28	–
**DSG4**	29789445	V	262	I	3Q2V_B	Ca^2+^	0.42	0.00
**GZMH**	15529990	V	207	M	1XXF_B	Na^+^	0.41	0.11
**HPCAL4**	7705419	P	10	H	1G8I_B	Na^+^	1.17	0.00
**LCT**	32481206	V	565	E	2ZOX_A	Mg^2+^	1.27	2.72
**LMX1A**	28893581	C	62	Y	2XJY_A	Zn^2+^	0.82	−3.23
**MAPK9**	21237736	G	35	R	1JNK_A	Mg^2+^	2.02	−1.06, −1.38
**NELL2**	5453766	D	602	N	1UZJ_B	Ca^2+^	0.78	0.27
**SGK2**	20127541	R	152	G	3KGA_A	Mg^2+^	2.02	0.01
**TP53**	120407068	C	176	Y,F	1TSR_A	Zn^2+^	0.82,0.61	−4.72, −4.67
**TP53**	120407068	H	178	Q	1TSR_A	Zn^2+^	0.732	0.00
**TP53**	120407068	H	179	Y	1TSR_A	Zn^2+^	1.037	−5.25
**ZIK1**	77736604	T	393	A	1MEY_G	Zn^2+^	0.55	0.60
**ZNF497**	333033771	D	278	N	1U85_A	Zn^2+^	0.78	−0.02
**ZNF497**	333033771	Q	433	L	1NJQ_A	Zn^2+^	1.26	−0.84

Gene and protein identifiers are shown together with the PDB code of structural evidence of interactions (structure of homologous complex), physico-chemical distances between substituted amino scids (“Dist Phys”) and difference in binding energy (“dddG”). Several multiple substitutions of the same site are listed on the same line.

In general, substitutions with amino acids that have similar physico-chemical properties may not drastically alter the stability of a single protein or a complex. We calculated physico-chemical distances between wild-type and substituted residues and compared it with the binding energy difference for all protein complexes and their models (see Methods). The physico-chemical distance was defined as the Euclidean distance using ten different physico-chemical properties of amino acids [28]. As indicated by its corresponding *ΔΔΔG*, the effect of substitutions was statistically significantly correlated with the physico-chemical distance (Figure S1B in File S1, Pearson r_P_ = −0.50, p-value = 0.015). Specifically, large distances corresponded to large negative *ΔΔΔG* and *vice versa*, suggesting that substitutions of amino acids with very different properties are usually destabilizing. In turn, small changes in amino acid properties may result in additional stabilization of complexes. All the data about physico-chemical distances and effects on binding energy are available at ftp://ftp.ncbi.nih.gov/pub/panch/GBM/.

Such results also prompted us to estimate the potential amplitude of the effect of mutations even if structures or models were unavailable. Therefore, we calculated physico-chemical distances for all 695 mutations from 598 genes. We observed that distributions of physico-chemical distances that referred to amino acid substitutions on all types of interfaces, and protein-protein interfaces in particular, had significantly larger distances compared to non-interface regions (Figure 2B, p-value = 0.011, Wilcoxon test). For example, we observed that the first peak in Figure 2B around 0.5 mostly referred to substitutions of aliphatic residues into each other or aliphatic into polar residues with Val->Met being the most frequent. The substitutions of arginine and cysteine were among the most frequent, had physico-chemical distances of about 1÷1.5 and corresponded to the second peak of the distribution in Figure 2B. In addition, we found that mutations often affected arginine on binding interfaces. Arginine has unique binding properties originating from strong stabilization of its protonated form due to its high pKa. Furthermore, Arg forms salt bridges, strong cation-π interactions and is enriched in binding hot spots [35], [36], [37].

### Interface Analysis Complements Machine-learning Methods and Helps to Decipher Molecular Mechanisms

Several machine-learning methods were recently developed to predict the phenotypic effect of disease mutations on proteins and were successfully used for monogenic diseases [5], [6]. Most of these methods utilize evolutionary conservation, residue mutability and accessible surface area as their main predictive features. We predicted the effect for 581 glioblastoma mutations using PolyPhen2, performing quite well in comparison to other prediction methods [38]. Our results showed that PolyPhen predicted 69% of all mutations on interfaces as “probably damaging” (Table S2 in File S1, tables at ftp://ftp.ncbi.nih.gov/pub/panch/GBM/). Such an agreement is noteworthy, given that our protocol is not trained on a known set of disease mutations while methods like PolyPhen do not use interface features in their training. Interestingly, we also found a limited but still significant correlation between the largest absolute value of the energetic effect of mutations on protein binding *ΔΔΔG* (as obtained by our approach) and the corresponding PolyPhen2 score (Spearman rank correlation, r_S_ = 0.5, p-value = 0.03). Since 23% of all mutations that PolyPhen predicted as “probably damaging” were located on interfaces, our approach may suggest the possible mechanism of their damaging effect through their impact on protein interactions.

As previously noted, many machine learning methods assessing the effects of mutations erroneously predict a ‘benign’ effect when the mutation occurs in an evolutionarily non-conserved position or is solvent accessible [9]. Yet, when a protein complex is formed, a mutation which was previously solvent accessible in a monomer (and possibly non-conserved) may be buried on the binding interface and very damaging for protein interactions. We show that 18% of interface mutations were predicted by Polyphen2 as “benign”; we analyze their possible driver effect and mechanism of action in the next two sections. Therefore, the necessity to develop approaches which complement machine learning methods with more detailed biophysical analyses is evident and should be the subject of future endeavors.

### Mechanisms of Effects of Mutations on Protein-protein Interactions

Here, we analyze the effect of mutations on protein-protein complexes and suggest the underlying mechanisms which include inactivation of wild-type enzymatic activity, destabilization of a functional multimeric complex and alteration of the protein turnover rate. All analyzed mutations were predicted to be benign by PolyPhen2. The first case represents the *IDH1* R132H mutation potentially inactivating the wild type conversion of isocitrate to α-ketoglutarate (α-KG) and/or resulting in a neo-enzymatic activity and production of D-2-hydroxyglutarate [39]. Since *IDH1* mutations are heterozygous we first analyzed the heterodimer containing one mutated and one wild type chain. Specifically, we found that heterodimers in the inactive state of *IDH1* (PDB code 1T09) were considerably stabilized by 8.6 kcal/mol. In addition, we performed calculations for a double mutant where both chains contained the R132H mutation, and showed that its inactive dimer is further stabilized by 11.3 kcal/mol. Our results are consistent with former studies suggesting that *IDH1* heterodimers are stable with a considerably lowered isocitrate dehydrogenase activity while R132H:R132H homodimers were almost completely inactive [40]. In accordance with other experimental studies, we suggest that such inactive dimer over-stabilization might prevent the conformational cooperative movements of dimer subunits required to form the active state [41].

Neuroligins (NLs) are transmembrane proteins on the postsynaptic cell surface and serve as receptors for neurexins that are synaptic cell adhesion proteins on the presynaptic cell surface. Since the formation of proper synapses is crucial for normal brain function we investigated the model of neuroligin2 (*NLGN2*) based on the neuroligin-1/neurexin-1 beta complex [42] (PDB code 3BIW, 75% identity between *NLGN2* and structural template). Earlier it was determined that the synaptogenic activity strongly depended on the formation of stable neuroligin-1 multimers [43]. We observed that glioblastoma mutation E577K was located on the dimer interface of two neuroligin monomers and contributed to a destabilization of this dimer by 1.2 kcal/mol [43].

The third example represents Rad52 playing a critical role in DNA double-strand-break repair. This protein is characterized by a very rapid turnover that is tightly regulated in the cell. Specifically, we observed that mutation R46K was located on the multimeric interface in the model of RAD52 N-terminal half of the protein (pdb code 1KN0) and considerably over-stabilized each dimer in the undecameric complex by 9 kcal/mol. Such a result might suggest that this mutation may considerably affect the Rad52 turnover rate. Indeed, it was previously shown that some mutants extend the half-life of Rad52 and dysregulate their turnover in a cell [44].

### Mechanisms of Effects of Mutations on Protein-nucleic Acid and Protein-ion Interfaces

**Figure 3:**
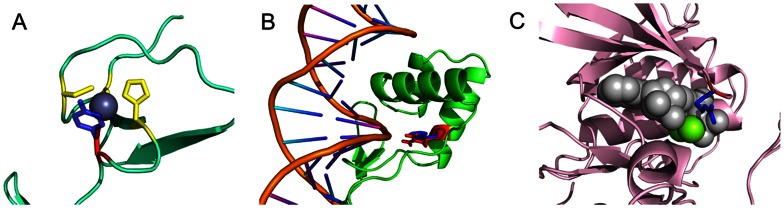
Examples of mutations on protein-nucleic acid and protein-ion interactions. Residues at mutated sites on homologous proteins are shown in red (wild type) and blue (mutant) stick models. (**A**) Zinc binding motif of LMO-2, homologous protein of *LMX1A* (PDB: 2XJY chain A, sequence identity 35%). A zinc ion is shown as a dark blue sphere. Zinc binding residues are shown in yellow stick models. (**B**) DNA binding site of *Pax-6*, homolog of *Pax-9* (PDB: 6PAX chain A, sequence identity: 74%). (**C**) *MAPK10*, homolog of *MAPK9*, with Mg-ANP (ATP analog) (PDB: 1JNK chain A, sequence identity: 85%). Mg ions are shown as green spheres and ANP is shown using a white sphere representation.

Other than protein-protein interactions, cancer mutations may affect other types of protein interactions as well. Altogether we found 16 and 13 mutations mapped to protein-ion and protein-nucleic acid binding interfaces, respectively. Table 1 shows representative examples with mutations located on binding interfaces and lists candidates for cancer biomarkers. As indicated in Table 1, mutations on five genes that correspond to protein-DNA or protein-ion interactions (*BCL11A, ZIK1, ZNF497, ZNF339*, and *TP53*) are located within C2H2-type zinc finger motifs. The zinc ion is essential for the stabilization of the local structure required for DNA binding. The disruption of Zn ion coordination may potentially lead to deregulation of corresponding proteins. Specifically, we found a C62Y substitution in LIM homeobox transcription factor 1 alpha (*LMX1A*), an important factor for the development of the nervous system. This transcription factor harbors two LIM zinc-binding domains, and the C62Y substitution occurs at one of the zinc-binding cysteine residues in the structure of its homolog LMO-2 and leads to decreased Zn-binding by 3.2 kcal/mol (see tables on ftp site) (Figure 3A). Indeed, a recent study suggested that *LMX1A* might play a tumor suppressive role and may be targeted for therapeutic intervention in human [45].

Paired box protein *PAX9* is another example originating from the transcription factor Pax family that regulates the expression of target genes involved in proliferation, stem-cell self-renewal, resistance to apoptosis and cell migration. *PAX9* expression is associated with favorable outcome in several cancers although its role in tumorigenesis is not well understood [46]. We studied the substitution R26W which, according to the crystal structure of its homolog *PAX6* (75% identical to *PAX9*), directly interacts with the DNA molecule [47] (Figure 3B). Although the substitution with tryptophan may have rather drastic consequences for the maintenance of the networks of electrostatic interactions between arginine and DNA phosphates, we did not find considerable differences in protein-DNA binding affinity (*ΔΔΔG* = –0.05 kcal/mol) although the mutation destabilizes an overall complex by 2.15 kcal/mol.

Cancer mutations may also directly affect enzymatic activity. Being involved in proliferation, differentiation and apoptosis pathways, mitogen-activated protein kinase 9 (*MAPK9*) blocks the ubiquitination of tumor suppressor p53 leading to an increase of suppressor stability. Similar to other phosphate transferring enzymes, *MAPK9* uses magnesium as a cofactor for phosphorylation. We studied G35R substitution in *MAPK9* and hypothesized that it might disrupt its tumor suppressor properties. The crystal structure of its homolog *MAPK10* (with 85% sequence identity to MAPK9) shows that Gly35 is located at the edge of the ATP binding pocket and participates in an ATP-binding loop [48]. According to FoldX calculations the substitution of glycine into positively charged arginine compromises magnesium cation binding by 1.38 kcal/mol, supporting the deregulation of *MAPK9* kinase activity and cancer cell development (Figure 3C).

### Properties of Mutated Interaction Network

**Figure 4:**
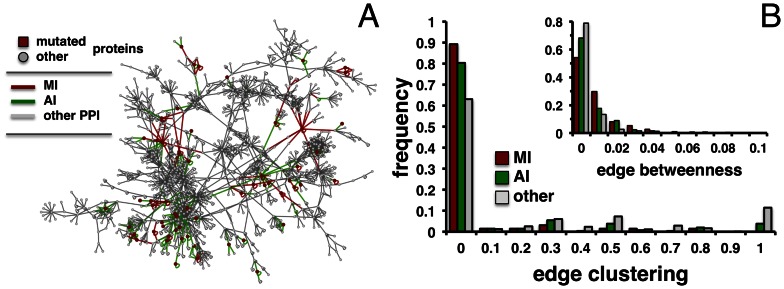
Network analysis of protein interactions that are affected by mutations. (**A**) By mapping all structurally inferred interactions that suffer from a mutation on their interfaces in a human interaction network (MI) we obtained a largest component capturing 1,960 interactions. Furthermore, we indicated all interactions that involved a mutated protein (AI). (**B**) Calculating edge clustering of MI and AI interactions in the largest component, we observed that interactions affected by a mutation generally tend to appear in less clustered areas. Compared to the remaining interactions, such differences were significant for both MI and AI interactions (p-value = 5×10^−8^, Wilcoxon test). Comparing MI and AI interactions, we observed a significant shift of MI interactions toward lower clustering (p-value = 0.01). In the inset, we determined edge betweenness of MI and AI interactions as a measure of their centrality in the network. Compared to the remaining interactions, we found that differences between both sets of interactions affected by mutations were statistically significant (p-value = 5×10^−3^). Furthermore, MI showed significantly lower betweenness than AI interactions (p-value = 0.01).

Topological network analysis facilitates the interpretation of interaction data and may allow the inference of cellular functions from the underlying proteins [49]. By mapping mutations and corresponding substitutions on protein-protein interfaces using our IBIS structural inference approach [50], [51], we identified 160 protein-protein interactions between 150 proteins with mutations that were located directly on binding interfaces (“mutant interactions”, MI). Furthermore, we embedded these interactions in a web of 4,073 interactions between 2,928 human proteins where each interaction was obtained by high-throughput methods as well as confirmed by the IBIS structural inference approach. In such a ‘confirmed interaction network’ we considered interactions that involved a protein with a mutation anywhere in a protein, allowing us to collect 444 “all mutant interactions” (AI). Therefore, the set of MI interactions is a subset of the AI set. To determine the role that “MI” and “AI” interactions play in a large human interaction network we determined the topological characteristics of such affected interaction networks. While we observed that the confirmed interaction network breaks into many connected components, we carried out our topological investigations on the largest connected component of 1,960 interactions (Figure 4A).

As a measure of clustering around a given interaction, we defined the edge clustering coefficient [52]. Assuming that MI interactions play a critical role in the flow of biological information in an interaction network, we hypothesized that such interactions may not be necessarily clustered but tend to bridge clustered areas. Indeed, we observed that MI and AI interactions generally tend to be placed in less clustered areas compared to the remaining unaffected interactions in Figure 4B (Wilcoxon test, p-value = 5×10^−8^). Notably, we also observed a significant shift to lower clustering of MI interactions compared to AI interactions (p-value = 0.01). A measure of an interaction’s centrality in a network is its edge betweenness centrality. Specifically, edge betweenness centrality determines the number of shortest paths through a given edge, therefore corresponding to potential “bottlenecks”. In the inset of Figure 4B, we show that interactions between proteins with mutations on binding interfaces (MI) had a significantly higher betweenness centrality than interactions involving non-mutant proteins (p-value = 0.005). We also found that such interactions had significantly higher betweenness than interactions between mutant proteins where mutation did not necessarily affect the binding interface (AI) (p-value = 0.01).

### Conclusions

Many studies have shown that missense mutations might play a very important role in causing different diseases. However, causal variants and phenotypic effects of these mutations are very difficult to predict, especially for polygenic diseases [53]. Although mutations of monogenic diseases might prefer the core of the protein [54], cancer related mutations exhibit quite a different pattern. Specifically, such mutations are less likely to occur in the protein core and prefer binding interfaces [20], [23]. Nevertheless the extent to which mutations might affect biomolecular interactions remains largely unknown. With this goal in mind we addressed the molecular mechanism of carcinogenic effects of glioblastoma mutations. The actual placement of cancer mutations on binding interfaces allowed us to stratify cancer-related interactions and potential driver genes and address the ways such mutations may affect binding and the underlying protein interaction network’s topology.

First, we found that overall missense mutations had a significantly destabilizing effect on protein-protein interactions although some mutations over-stabilized protein complexes. This effect was mostly driven by the electrostatic component of binding energy and such observations are consistent with previous investigations, focusing on the effects of OMIM mutations on protein complexes [22]. Indeed, the charge complementarity may determine specific binding while its disruption can be accompanied by the loss of specific interactions. The contribution of a given charged pair of amino acids to the electrostatic component of binding energy depends on the balance of two large terms: desolvation penalty and electrostatic pairwise interactions. While the desolvation penalty of a group mostly depends on its net charge, the pairwise electrostatic interaction energy is also sensitive to the geometry of the side chains. Previous results indicated that electrostatic interactions on protein-protein binding interfaces are almost always favorable [55]. Therefore, amino acid substitutions might result in dramatic changes of the magnitude of the favorable pairwise electrostatic interactions, while having little impact on the desolvation penalty [56].

Furthermore, we calculated changes in physico-chemical properties between wild-type and substituted residues and found that amino acid substitutions on binding interfaces were associated with significantly larger physico-chemical distances compared to non-interface mutations. The most drastic amino acid changes were observed for mutations on protein-protein binding interfaces. Such observations point to the possible damaging effect of many glioblastoma mutations on interfaces. We expect that such mutations are unlikely passenger mutations but rather may drive the disease phenotype. Such an argument appears plausible given that genetic alterations in cancer generally affect signaling pathways, compromising many protein-protein interaction events. Specifically, we detected a group of potential cancer biomarkers, some of them specific to nervous system development, which might be deregulated by mutations affecting protein-protein, protein-nucleic acid and protein-ion binding.

Since many factors influence the topology of protein interaction networks and the role of a given protein in cancer development, a debate about the topological properties of disease and cancer-related networks has emerged. Although the vast majority of disease genes were found to be nonessential and did not encode hub proteins, cancer related genes, however, suggested an opposite trend [16], [57], [58]. High betweenness of proteins that translates into being a “bottleneck” indicated another important characteristic that distinguished essential and non-essential genes especially in regulatory networks [59]. Hub-bottlenecks, for example, defined as frequently interacting proteins connecting different clustered areas, were indicated as good predictors of gene essentiality [16]. Here we found that interactions involving proteins with mutations generally tend to occur in less clustered areas and are characterized by higher edge betweenness compared to the remaining unaffected interactions from the same network. Importantly, we also show that our stratification of cancer-relevant interactions had a significant impact by focusing on glioblastoma mutations observed directly on binding interfaces (MI). In particular, MI interactions were placed in areas of lower clustering and higher edge betweenness compared to AI interactions that did not account for the mutated position with respect to protein binding. Still, AI interactions had higher bottleneck properties compared to the remaining, unaffected interactions.

Indeed, although oncogenes were reported to have a certain tendency to cluster into a small number of modules and pathways [60], [61], other reports did not confirm such characteristics [16]. Here, we show that genes with mutations affecting their binding interfaces were preferably located in central network positions which might influence critical nodes/edges in signal transduction networks mediated by protein-protein interactions. Our observation is consistent with two previous studies, indicating that proteins extensively involved in signal transduction activity, actively sending and receiving signals, are more frequently mutated in cancer [60].

## Materials and Methods

### Constructing Human Interactome and Mapping Mutations on Binding Interfaces

We used our recently developed framework to map the human interactome [18]. Selecting the longest protein isoforms of a human query sequence, we retrieved their protein interaction partners and binding sites using the IBIS server (http://www.ncbi.nlm.nih.gov/Structure/ibis/ibis.cgi) [50], [51]. IBIS predicts protein interaction partners and provides the locations of their binding sites on a query protein using a set of homologous structural complexes as evidence of an interaction. Along with different types of protein interaction partners (protein, ion, DNA, RNA, peptide, and small molecule), IBIS ensures the biological relevance of binding sites. Utilizing structural complexes, IBIS collects experimentally ‘observed’ protein interactions if a protein has a certain number of residues that ‘contact’ its partner. Two residues are considered to be in contact if any of the heavy-atom inter-atomic distances is less than 6 Å for protein-protein (4 Å for protein-nucleic acid and 3 Å for protein-ion) interaction partners. Such a group of residues that is in contact to an interaction partner is called a “*binding site*”. Demanding that the sequence similarity between the query protein and a homologous structural complex is high enough (see Supporting Information for details), homologous complexes were subsequently grouped according to their binding site similarity. In the case of protein-protein interactions we mapped interaction partners from complexes of other organisms to their most similar human proteins that had more than 80% sequence identity and 80% protein sequence coverage. As a result of this procedure we obtained 54,861 protein-protein interactions between 9,265 human proteins, a network we refer to as ‘structurally inferred’. In addition, we used IBIS to determine protein-nucleic acids and protein-ion interactions, procedures that did not require additional mapping of the interactions partners to human proteins.

We also pooled 61,240 interactions between 11,446 proteins from high-throughput experiments found in Reactome [62], MINT [63] and HPRD [64]. We confirmed these interactions by using structurally inferred interactions, allowing us to obtain 4,073 interactions between 2,928 human proteins, a network that we refer to as the “confirmed interaction network”. We compiled missense mutations from genes in glioblastoma multiform patients from two previous studies [25], [26] that identified mutations in the tumor sequences that were not present in the reference sequences of each gene. Mutations present in the normal control samples and in single nucleotide polymorphism (SNP) databases were then removed from further analyses. We additionally verified interaction interfaces in complexes using the PISA algorithm. Using chemical thermodynamics, PISA computes a set of macromolecular assemblies that are expected to be stable in solution and are assumed to represent the biological form of a protein in the cell [65]. In the end, we mapped 695 missense mutations from 598 genes on different types of proteins and protein binding interfaces as shown in Figure 1. Altogether 97 mutations from 68 genes were mapped on protein-protein interfaces affecting 160 protein-protein interactions, whereas 16 and 13 mutations were found on protein-ion and protein-nucleic acid interfaces respectively (ftp://ftp.ncbi.nih.gov/pub/panch/GBM/). Among those mutations mapped on interfaces, 33% mutations were observed in more than one cancer sample according to the COSMIC database [66] compared to 15% of mutations which could not be mapped on interfaces.

### Modeling of Protein Complexes

Based on templates identified in IBIS we built 3D structural models of protein-protein complexes using a homology modeling approach. In particular, we applied the Profix and Nest programs provided by the Jackal package [67] to fix missing atoms or residues of the templates and to build homology models of complexes based on their sequence alignments. The models were submitted to the TINKER package for energy minimization utilizing the Limited Memory BFGS Quasi-Newton Optimization algorithm [68]. Energy minimization was performed with the Amber98 force field. The convergence criterion was set to the root mean-squared (RMS) gradient per atom = 0.01. The Scap program [69] as provided by the Jackal package was applied to computationally generate the corresponding mutant structures using the minimized wild-type models, and the mutations were introduced by side-chain replacements. The wild-type and mutant structures were minimized again using TINKER to assure that both structures were subjected to the same refinement protocol after introducing the mutation. It was previously shown that state-of-the-art algorithms can always build high-quality models for proteins with sequence identity higher than 35∼40% to the homologous protein structures [70]. Checking the quality of the models, we removed models with large van-der-Waals clashes based on calculated energies and selected only those models that were based on protein-protein structural complexes with more than 40% identity to both the query human protein and its interaction partner which resulted in 21 high quality models of protein-protein complexes. Protein-DNA and protein-ion complexes were modeled using Modeller [71] “automodel” function. Mutations were introduced using FoldX “buildmodel” module. DNA molecules were treated as non-flexible during the modeling processes.

### Binding Energy Calculation

Binding energy was calculated using the rigid body approach described in previous studies [22], [72], [73]. We assumed that the internal mechanical energies of the (un-)bound monomers remain unchanged so that the energy terms of the unfolded state can be excluded in the binding energy calculation. The total potential energy and its two components, van der Waals energy and electrostatic energy, for protein-protein complexes were computed with the ANALYZE program as provided by the TINKER package [68]. The electrostatic component of total potential energy was defined as the sum of charge-charge interaction energy and continuum solvation energy obtained from the TINKER results. The binding energy was defined as the difference between the potential energy of the dimer and the individual monomers.
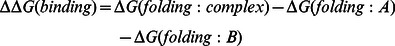
(1)where Δ*G(folding: complex)*, Δ*G(folding: A)* and Δ*G(folding: B)* were the potential energies of the dimer complex, monomer A and monomer B, respectively.

The effect of a mutation on binding affinity was assessed by using the binding energy difference between the wild-type (WT) and mutant structure (MU):
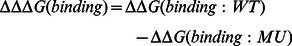
(2)where ΔΔ*G*(binding: WT) and ΔΔ*G*(binding: MU) were the binding energies of the wild-type (WT) complex and the mutant complex (MU) calculated using equation (1). The difference of total potential energy *(*ΔΔΔ*G_tot_),* van der Waals energy *(*ΔΔΔ*G_vdW_)* and electrostatic energy *(*ΔΔΔ*G_el_)* were calculated and analyzed separately. A negative value of ΔΔΔ*G_ binding_* indicated that the mutation decreased the binding affinity, destabilizing the complex. In turn, a positive value of ΔΔΔ*G_ binding_* suggested that the mutation stabilized the complex.

We also used the “AnalyseComplex” function of the FoldX program [74] which calculates the stability of protein complexes using an empirical force field to estimate the effect of mutations on binding. The change in binding energy was approximated by impairing the selected targets, determining the stability of the separated molecules and then subtracting the sum of the individual energies from the global energy of the complex. Binding energy differences for protein-DNA interactions were calculated similarly. In these cases *ΔG(folding: complex)* refers to the stability of the protein-DNA complex and *ΔG(folding: A)* refers to the stability of the monomer without DNA. Binding energy of protein-ion interactions were calculated by “MetalBindingEnergy” function of FoldX. The best five models (with the highest Modeller molpdf score) were analyzed and the mean values of ΔΔΔ*G* calculated. All protein-DNA and protein-ion models were preprocessed by the RepairPDB function of FoldX before any energy calculation to optimize side chain conformation.

### Topological Measures of Interaction Networks

As a global measure of a protein-protein interaction’s centrality, we calculated edge betweenness, reflecting an edge’s appearance in shortest paths through the whole network. In particular, we defined edge betweenness centrality of an interaction *v* as 
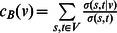
. *σ(s,t)* was the number of shortest paths between proteins *s* and *t* while *σ(s,t|v)* was the number of shortest paths through edge *v*. Furthermore, we normalized edge betweenness values by 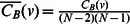 where *N* was the number of proteins in the connected component in which the underlying edge appeared. The edge clustering coefficient of interacting proteins *i* and *j* was defined as 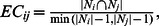 where *N_i_* and *N_j_* were the sets of interaction partners of *i* and *j*, respectively [52].

### Statistical Analysis

To perform statistical tests we used Splus and R packages. To compare mean values of two distributions (or compare the mean value to zero), we used the Wilcoxon rank test and reported one-sided p-values. To test the independence between the rows and columns in a contingency table, we used Fisher’s exact test and reported two-sided p-values. We also calculated Pearson and Spearman correlation coefficients to test the null hypothesis about the independence of two variables and reported two-sided p-values.

## Supporting Information

File S1 This file contains Table S1 (Phosphorylation of mutated sites), Table S2 (Prediction of the effect of mutations on protein-protein and non-interface regions using PolyPhen2), Figure S1 (The change of binding energy) and Text S1 (Mapping of complete interactomes using structural complexes, methodology).
(DOCX)

## References

[1] Loeb LA (1991) Mutator phenotype may be required for multistage carcinogenesis. Cancer Res 51: 3075–3079. 2039987

[2] Weinstein IB, Joe A (2008) Oncogene addiction. Cancer Res 68: 3077–3080; discussion 3080. 10.1158/0008-5472.CAN-07-3293 18451130

[3] Wood LD, Parsons DW, Jones S, Lin J, Sjoblom T, et al (2007) The genomic landscapes of human breast and colorectal cancers. Science 318: 1108–1113. 17932254 10.1126/science.1145720

[4] Greenman C, Stephens P, Smith R, Dalgliesh GL, Hunter C, et al (2007) Patterns of somatic mutation in human cancer genomes. Nature 446: 153–158. 17344846 10.1038/nature05610 PMC2712719

[5] Ng PC, Henikoff S (2003) SIFT: Predicting amino acid changes that affect protein function. Nucleic Acids Res 31: 3812–3814. 12824425 10.1093/nar/gkg509 PMC168916

[6] Adzhubei IA, Schmidt S, Peshkin L, Ramensky VE, Gerasimova A, et al (2010) A method and server for predicting damaging missense mutations. Nat Methods 7: 248–249. 20354512 10.1038/nmeth0410-248 PMC2855889

[7] Ding L, Getz G, Wheeler DA, Mardis ER, McLellan MD, et al (2008) Somatic mutations affect key pathways in lung adenocarcinoma. Nature 455: 1069–1075. 18948947 10.1038/nature07423 PMC2694412

[8] Youn A, Simon R (2011) Identifying cancer driver genes in tumor genome sequencing studies. Bioinformatics 27: 175–181. 21169372 10.1093/bioinformatics/btq630 PMC3018819

[9] Kumar S, Suleski MP, Markov GJ, Lawrence S, Marco A, et al (2009) Positional conservation and amino acids shape the correct diagnosis and population frequencies of benign and damaging personal amino acid mutations. Genome Res 19: 1562–1569. 19546171 10.1101/gr.091991.109 PMC2752122

[10] Torkamani A, Verkhivker G, Schork NJ (2009) Cancer driver mutations in protein kinase genes. Cancer Lett 281: 117–127. 19081671 10.1016/j.canlet.2008.11.008 PMC2905872

[11] Tennessen JA, Bigham AW, O'Connor TD, Fu W, Kenny EE, et al (2012) Evolution and functional impact of rare coding variation from deep sequencing of human exomes. Science 337: 64–69. 22604720 10.1126/science.1219240 PMC3708544

[12] Cerami E, Demir E, Schultz N, Taylor BS, Sander C (2010) Automated network analysis identifies core pathways in glioblastoma. PLoS One 5: e8918. 20169195 10.1371/journal.pone.0008918 PMC2820542

[13] Hosur R, Xu J, Bienkowska J, Berger B (2011) iWRAP: An interface threading approach with application to prediction of cancer-related protein-protein interactions. J Mol Biol 405: 1295–1310. 21130772 10.1016/j.jmb.2010.11.025 PMC3028939

[14] Kim YA, Wuchty S, Przytycka TM (2011) Identifying causal genes and dysregulated pathways in complex diseases. PLoS Comput Biol 7: e1001095. 21390271 10.1371/journal.pcbi.1001095 PMC3048384

[15] Torkamani A, Schork NJ (2009) Identification of rare cancer driver mutations by network reconstruction. Genome Res 19: 1570–1578. 19574499 10.1101/gr.092833.109 PMC2752121

[16] Kar G, Gursoy A, Keskin O (2009) Human cancer protein-protein interaction network: a structural perspective. PLoS Comput Biol 5: e1000601. 20011507 10.1371/journal.pcbi.1000601 PMC2785480

[17] Huang YJ, Hang D, Lu LJ, Tong L, Gerstein MB, et al (2008) Targeting the human cancer pathway protein interaction network by structural genomics. Mol Cell Proteomics 7: 2048–2060. 18487680 10.1074/mcp.M700550-MCP200 PMC2559933

[18] Tyagi M, Hashimoto K, Shoemaker BA, Wuchty S, Panchenko AR (2012) Large-scale mapping of human protein interactome using structural complexes. EMBO Rep 13: 266–271. 22261719 10.1038/embor.2011.261 PMC3296913

[19] Kuzu G, Keskin O, Gursoy A, Nussinov R (2012) Constructing structural networks of signaling pathways on the proteome scale. Curr Opin Struct Biol 22: 367–377. 22575757 10.1016/j.sbi.2012.04.004

[20] Wang X, Wei X, Thijssen B, Das J, Lipkin SM, et al (2012) Three-dimensional reconstruction of protein networks provides insight into human genetic disease. Nat Biotechnol 30: 159–164. 22252508 10.1038/nbt.2106 PMC3708476

[21] Schuster-Bockler B, Bateman A (2008) Protein interactions in human genetic diseases. Genome Biol 9: R9. 18199329 10.1186/gb-2008-9-1-r9 PMC2395246

[22] Teng S, Madej T, Panchenko A, Alexov E (2009) Modeling effects of human single nucleotide polymorphisms on protein-protein interactions. Biophys J 96: 2178–2188. 19289044 10.1016/j.bpj.2008.12.3904 PMC2717281

[23] David A, Razali R, Wass MN, Sternberg MJ (2012) Protein-protein interaction sites are hot spots for disease-associated nonsynonymous SNPs. Hum Mutat 33: 359–363. 22072597 10.1002/humu.21656

[24] Kleihues P, Louis DN, Scheithauer BW, Rorke LB, Reifenberger G, et al.. (2002) The WHO classification of tumors of the nervous system. J Neuropathol Exp Neurol 61: 215–225; discussion 226–219. 10.1093/jnen/61.3.215 11895036

[25] Network TCGAR (2008) Comprehensive genomic characterization defines human glioblastoma genes and core pathways. Nature 455: 1061–1068. 18772890 10.1038/nature07385 PMC2671642

[26] Parsons DW, Jones S, Zhang X, Lin JC, Leary RJ, et al (2008) An integrated genomic analysis of human glioblastoma multiforme. Science 321: 1807–1812. 18772396 10.1126/science.1164382 PMC2820389

[27] Ohgaki H, Kleihues P (2009) Genetic alterations and signaling pathways in the evolution of gliomas. Cancer Sci 100: 2235–2241. 19737147 10.1111/j.1349-7006.2009.01308.x PMC11159448

[28] Kawashima S, Pokarowski P, Pokarowska M, Kolinski A, Katayama T, et al (2008) AAindex: amino acid index database, progress report 2008. Nucleic Acids Res 36: D202–205. 17998252 10.1093/nar/gkm998 PMC2238890

[29] Radivojac P, Baenziger PH, Kann MG, Mort ME, Hahn MW, et al (2008) Gain and loss of phosphorylation sites in human cancer. Bioinformatics 24: i241–247. 10.1093/bioinformatics/btn267 PMC2732209 18689832

[30] Hornbeck PV, Chabra I, Kornhauser JM, Skrzypek E, Zhang B (2004) PhosphoSite: A bioinformatics resource dedicated to physiological protein phosphorylation. Proteomics 4: 1551–1561. 15174125 10.1002/pmic.200300772

[31] Dinkel H, Chica C, Via A, Gould CM, Jensen LJ, et al (2011) Phospho.ELM: a database of phosphorylation sites–update 2011. Nucleic Acids Res 39: D261–267. 21062810 10.1093/nar/gkq1104 PMC3013696

[32] Gnad F, Gunawardena J, Mann M (2011) PHOSIDA 2011: the posttranslational modification database. Nucleic Acids Res 39: D253–260. 21081558 10.1093/nar/gkq1159 PMC3013726

[33] Xue Y, Liu Z, Cao J, Ma Q, Gao X, et al (2011) GPS 2.1: enhanced prediction of kinase-specific phosphorylation sites with an algorithm of motif length selection. Protein Eng Des Sel 24: 255–260. 21062758 10.1093/protein/gzq094

[34] Nishi H, Hashimoto K, Panchenko AR (2011) Phosphorylation in protein-protein binding: effect on stability and function. Structure 19: 1807–1815. 22153503 10.1016/j.str.2011.09.021 PMC3240861

[35] Bogan AA, Thorn KS (1998) Anatomy of hot spots in protein interfaces. J Mol Biol 280: 1–9. 9653027 10.1006/jmbi.1998.1843

[36] Tyagi M, Shoemaker BA, Bryant SH, Panchenko AR (2009) Exploring functional roles of multibinding protein interfaces. Protein Sci 18: 1674–1683. 19591200 10.1002/pro.181 PMC2776955

[37] Tuncbag N, Gursoy A, Keskin O (2009) Identification of computational hot spots in protein interfaces: combining solvent accessibility and inter-residue potentials improves the accuracy. Bioinformatics 25: 1513–1520. 19357097 10.1093/bioinformatics/btp240

[38] Wei Q, Wang L, Wang Q, Kruger WD, Dunbrack RL Jr (2010) Testing computational prediction of missense mutation phenotypes: functional characterization of 204 mutations of human cystathionine beta synthase. Proteins 78: 2058–2074. 20455263 10.1002/prot.22722 PMC3040297

[39] Dang L, White DW, Gross S, Bennett BD, Bittinger MA, et al (2009) Cancer-associated IDH1 mutations produce 2-hydroxyglutarate. Nature 462: 739–744. 19935646 10.1038/nature08617 PMC2818760

[40] Zhao S, Lin Y, Xu W, Jiang W, Zha Z, et al (2009) Glioma-derived mutations in IDH1 dominantly inhibit IDH1 catalytic activity and induce HIF-1alpha. Science 324: 261–265. 19359588 10.1126/science.1170944 PMC3251015

[41] Xu X, Zhao J, Xu Z, Peng B, Huang Q, et al (2004) Structures of human cytosolic NADP-dependent isocitrate dehydrogenase reveal a novel self-regulatory mechanism of activity. J Biol Chem 279: 33946–33957. 15173171 10.1074/jbc.M404298200

[42] Arac D, Boucard AA, Ozkan E, Strop P, Newell E, et al (2007) Structures of neuroligin-1 and the neuroligin-1/neurexin-1 beta complex reveal specific protein-protein and protein-Ca2+ interactions. Neuron 56: 992–1003. 18093522 10.1016/j.neuron.2007.12.002

[43] Dean C, Scholl FG, Choih J, DeMaria S, Berger J, et al (2003) Neurexin mediates the assembly of presynaptic terminals. Nat Neurosci 6: 708–716. 12796785 10.1038/nn1074 PMC1646425

[44] Asleson EN, Livingston DM (2003) Investigation of the stability of yeast rad52 mutant proteins uncovers post-translational and transcriptional regulation of Rad52p. Genetics 163: 91–101. 12586699 10.1093/genetics/163.1.91 PMC1462433

[45] Dong W, Feng L, Xie Y, Zhang H, Wu Y (2011) Hypermethylation-mediated reduction of LMX1A expression in gastric cancer. Cancer Sci 102: 361–366. 21159062 10.1111/j.1349-7006.2010.01804.x

[46] Robson EJ, He SJ, Eccles MR (2006) A PANorama of PAX genes in cancer and development. Nat Rev Cancer 6: 52–62. 16397527 10.1038/nrc1778

[47] Xu HE, Rould MA, Xu W, Epstein JA, Maas RL, et al (1999) Crystal structure of the human Pax6 paired domain-DNA complex reveals specific roles for the linker region and carboxy-terminal subdomain in DNA binding. Genes Dev 13: 1263–1275. 10346815 10.1101/gad.13.10.1263 PMC316729

[48] Xie X, Gu Y, Fox T, Coll JT, Fleming MA, et al (1998) Crystal structure of JNK3: a kinase implicated in neuronal apoptosis. Structure 6: 983–991. 9739089 10.1016/s0969-2126(98)00100-2

[49] Sharan R, Ideker T (2006) Modeling cellular machinery through biological network comparison. Nat Biotechnol 24: 427–433. 16601728 10.1038/nbt1196

[50] Shoemaker BA, Zhang D, Thangudu RR, Tyagi M, Fong JH, et al (2010) Inferred Biomolecular Interaction Server–a web server to analyze and predict protein interacting partners and binding sites. Nucleic Acids Res 38: D518–524. 19843613 10.1093/nar/gkp842 PMC2808861

[51] Shoemaker BA, Zhang D, Tyagi M, Thangudu RR, Fong JH, et al (2012) IBIS (Inferred Biomolecular Interaction Server) reports, predicts and integrates multiple types of conserved interactions for proteins. Nucleic Acids Res 40: D834–840. 22102591 10.1093/nar/gkr997 PMC3245142

[52] Wang J, Li M, Wang H, Pan Y (2011) Identification of Essential Proteins Based on Edge Clustering Coefficient. IEEE/ACM Trans Comput Biol Bioinform. 10.1109/TCBB.2011.147 22084147

[53] Stitziel NO, Kiezun A, Sunyaev S (2011) Computational and statistical approaches to analyzing variants identified by exome sequencing. Genome Biol 12: 227. 21920052 10.1186/gb-2011-12-9-227 PMC3308043

[54] Vitkup D, Sander C, Church GM (2003) The amino-acid mutational spectrum of human genetic disease. Genome Biol 4: R72. 14611658 10.1186/gb-2003-4-11-r72 PMC329120

[55] Brock K, Talley K, Coley K, Kundrotas P, Alexov E (2007) Optimization of electrostatic interactions in protein-protein complexes. Biophys J 93: 3340–3352. 17693468 10.1529/biophysj.107.112367 PMC2072065

[56] Selzer T, Albeck S, Schreiber G (2000) Rational design of faster associating and tighter binding protein complexes. Nat Struct Biol 7: 537–541. 10876236 10.1038/76744

[57] Jonsson PF, Bates PA (2006) Global topological features of cancer proteins in the human interactome. Bioinformatics 22: 2291–2297. 16844706 10.1093/bioinformatics/btl390 PMC1865486

[58] Goh KI, Cusick ME, Valle D, Childs B, Vidal M, et al (2007) The human disease network. Proc Natl Acad Sci U S A 104: 8685–8690. 17502601 10.1073/pnas.0701361104 PMC1885563

[59] Yu H, Kim PM, Sprecher E, Trifonov V, Gerstein M (2007) The importance of bottlenecks in protein networks: correlation with gene essentiality and expression dynamics. PLoS Comput Biol 3: e59. 17447836 10.1371/journal.pcbi.0030059 PMC1853125

[60] Cui Q, Ma Y, Jaramillo M, Bari H, Awan A, et al (2007) A map of human cancer signaling. Mol Syst Biol 3: 152. 18091723 10.1038/msb4100200 PMC2174632

[61] Wu G, Feng X, Stein L (2010) A human functional protein interaction network and its application to cancer data analysis. Genome Biol 11: R53. 20482850 10.1186/gb-2010-11-5-r53 PMC2898064

[62] Croft D, O'Kelly G, Wu G, Haw R, Gillespie M, et al (2011) Reactome: a database of reactions, pathways and biological processes. Nucleic Acids Res 39: D691–697. 21067998 10.1093/nar/gkq1018 PMC3013646

[63] Ceol A, Chatr Aryamontri A, Licata L, Peluso D, Briganti L, et al (2010) MINT, the molecular interaction database: 2009 update. Nucleic Acids Res 38: D532–539. 19897547 10.1093/nar/gkp983 PMC2808973

[64] Keshava Prasad TS, Goel R, Kandasamy K, Keerthikumar S, Kumar S, et al (2009) Human Protein Reference Database–2009 update. Nucleic Acids Res 37: D767–772. 18988627 10.1093/nar/gkn892 PMC2686490

[65] Krissinel E, Henrick K (2007) Inference of macromolecular assemblies from crystalline state. J Mol Biol 372: 774–797. 17681537 10.1016/j.jmb.2007.05.022

[66] Forbes SA, Bhamra G, Bamford S, Dawson E, Kok C, et al.. (2008) The Catalogue of Somatic Mutations in Cancer (COSMIC). Curr Protoc Hum Genet Chapter 10: Unit 10 11. 10.1002/0471142905.hg1011s57 PMC2705836 18428421

[67] Petrey D, Xiang Z, Tang CL, Xie L, Gimpelev M, et al (2003) Using multiple structure alignments, fast model building, and energetic analysis in fold recognition and homology modeling. Proteins 53 Suppl 6430–435. 14579332 10.1002/prot.10550

[68] Ponder JW (1999) TINKER-software tools for molecular design: St. Luis:Washington University.

[69] Xiang Z, Honig B (2001) Extending the accuracy limits of prediction for side-chain conformations. J Mol Biol 311: 421–430. 11478870 10.1006/jmbi.2001.4865

[70] Zhang Y (2009) Protein structure prediction: when is it useful? Curr Opin Struct Biol 19: 145–155. 19327982 10.1016/j.sbi.2009.02.005 PMC2673339

[71] Sali A, Blundell TL (1993) Comparative protein modelling by satisfaction of spatial restraints. J Mol Biol 234: 779–815. 8254673 10.1006/jmbi.1993.1626

[72] Teng S, Srivastava AK, Schwartz CE, Alexov E, Wang L (2010) Structural assessment of the effects of amino acid substitutions on protein stability and protein protein interaction. Int J Comput Biol Drug Des 3: 334–349. 21297231 10.1504/IJCBDD.2010.038396 PMC3319068

[73] Zhang Z, Teng S, Wang L, Schwartz CE, Alexov E (2010) Computational analysis of missense mutations causing Snyder-Robinson syndrome. Hum Mutat 31: 1043–1049. 20556796 10.1002/humu.21310 PMC2932761

[74] Guerois R, Nielsen JE, Serrano L (2002) Predicting changes in the stability of proteins and protein complexes: a study of more than 1000 mutations. J Mol Biol 320: 369–387. 12079393 10.1016/S0022-2836(02)00442-4

