# Supplementary material for: Cancer Missense Mutations Alter Binding Properties of Proteins and Their Interaction Networks
Source: PLoS One. 2013 Jun 14;8(6):e66273. doi: 10.1371/journal.pone.0066273 (PMC3682950; doi:10.1371/journal.pone.0066273)
Supplement: File S1 — This file contains Table S1 (Phosphorylation of mutated sites), Table S2 (Prediction of the effect of mutations on protein-protein and non-interface regions using PolyPhen2), Figure S1 (The change of binding energy) and Text S1 (Mapping of complete interactomes using structural complexes, methodology). (DOCX) [file pone.0066273.s001.docx]

**Supporting Information**

**Cancer missense mutations alter binding properties of proteins and their interaction networks**

Hafumi Nishi, Manoj Tyagi, Shaolei Teng, Benjamin Shoemaker, Kosuke Hashimoto, Emil Alexov, Stefan Wuchty and Anna R. Panchenko

**Table S1. Phosphorylation of mutated sites.** Phosphorylation sites are mapped on all Ser, Thr and Tyr residues of mutated proteins.

|  | phosphorylated | non-phosphorylated (S/T/Y) | total |
| --- | --- | --- | --- |
| mutated | 6 | 88 | 94 |
| non-mutated | 2819 | 112972 | 115791 |
| Total | 2825 | 113060 | 115885 |

**Table S2. Prediction of the effect of mutations on protein-protein and non-interface regions using PolyPhen2 (Adzhubei et al, 2010). Altogether Polyphen2 predictions were obtained for 581 mutations.**

| Predicted effect of mutation | Mutations on protein-protein interface | Mutations on all interactions | Mutations on non-interfaces |
| --- | --- | --- | --- |
| Benign | 16 | 17 | 188 |
| Possibly damaging | 8 | 12 | 81 |
| Probably damaging | 61 | 66 | 217 |


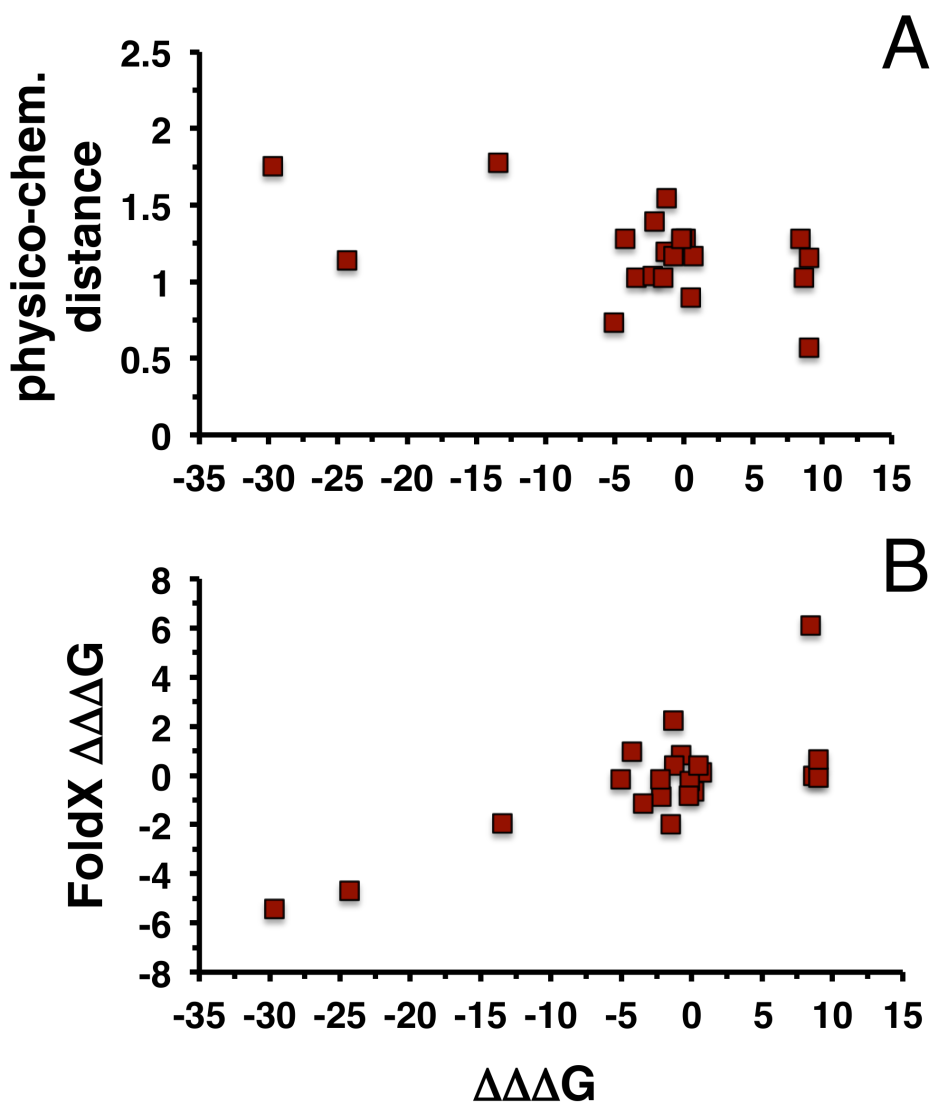


**Figure S1.** (A) The change of binding energy calculated by our protocol is plotted versus physico-chemical distance between substituted amino acids (Pearson’s r_P_ = -0.50, P = 0.02). (B) The changes of binding energy obtained by our protocol and FoldX respectively (Pearson’s r_P_ = 0.77, P = 10^-5^). The change in binding energy by FoldX was used with the minus sign.

**Text S1. Mapping of complete interactomes using structural complexes, methodology.**

The strength of our approach is that it ensures close evolutionary relationships between structural complexes and target proteins, and verifies the interactions and binding interfaces by several means. First, it examines the evolutionary conservation between homologous complexes under the assumption that if the binding site is conserved among non-redundant homologs, it is more likely to be biologically relevant (sites that are not conserved and lineage specific might be excluded unless they are very similar to the query based on the ranking score, see below). Second, our approach uses algorithms to infer correct biological units and, finally, it applies a

rigorous scheme to rank binding sites with respect to their relevance to the target protein. Such a ranking scheme includes sequence-PSSM score (where the Position Specific Scoring Matrix is constructed based on the alignment of binding sites from homologous complexes), overall sequence similarity between target protein and its homologs with known complexes, and the number of interface contacts. It should be mentioned that such a rigorous verification of interactions upon homology inference is essential since common descent does not necessarily imply similarity in function or interactions. Annotations transferred from one protein to a homolog may result in incorrect functional or interolog assignment at larger evolutionary distances, even for close homologs if they have different binding specificities. Since binding specificity is usually determined by the structural and sequence features of protein interaction interfaces, it is essential to detect and transfer binding sites correctly. To verify and guide predictions based on inference, one needs to ensure similarity between sites on the unknown target protein and on the conserved binding sites detected in homologs. Moreover, the PDB asymmetric unit which is usually used to infer interactions does not necessarily correspond to the biological state of a given protein. According to several studies (Jefferson et al, 2006; Xu et al, 2008), more than 20% of PDB complexes represent crystallographic packing errors and about 30% of PDB entries should be reconstructed by applying crystallographic symmetry operations.

**Major steps of the IBIS method for predicting protein interaction partners and binding site locations**

*-* ***Collecting homologs with observed interactions***

To infer interactions based on homology we first collected template proteins with known structures that are similar to a given query protein and have at least 80% sequence identity and more than 80% of the query sequence aligned using cBlast. For each template protein we retrieved all homologous (with more than 30% identity) and structurally-similar proteins with the known structural complexes from the Protein Data Bank. Template and homologous structural complexes were structurally aligned using the

VAST algorithm. Subsequently, homologous complexes were grouped based on their binding site similarity, assuming that a binding site is functionally important and is not lineage specific if it is evolutionarily conserved among non-redundant homologs.

*-* ***Measuring binding site similarity***

We cluster domain-binding sites into groups based on their sequence and structure similarity. The similarity score between two positions *i* and *j* of two binding sites is defined as (Thangudu et al, 2010):

| $S_{ij}=H\left( a_{i},a_{j} \right)\Delta_{ij}+\boldsymbol{\theta}\Delta_{ij}+w\left( 1-\Delta_{ij} \right)$ |  |
| --- | --- |
|  |  |

where *H* is the corresponding element of the BLOSUM62 matrix; *Δij* is equal to 1 if two positions are aligned and 0 otherwise; 𝜽 is an additional weight of “+1” added to each structurally equivalent position, and *w* is a gap penalty of “-4”. The overall similarity score between two binding sites is calculated by summing *Sij* over all positions in the gapped alignment.

*-* ***Clustering of binding sites***

The binding sites of the homologous structure neighbors are clustered using a complete-linkage clustering algorithm, which calculates the distance between two clusters as the maximum distance between their members. A distance cutoff value to define the clusters is chosen using a pseudo-free energy function from a study which maximizes the mean similarity of members within a cluster and minimizes the complexity of the description provided by cluster membership (Slonim et al, 2005). At the end of this procedure sets of binding residues (“binding sites”) from different homologs of the query protein are grouped together based on their similarity.

*-* ***Ranking of binding site clusters***

All binding site clusters are ranked in terms of biological relevance and similarity to the query. First, we check whether the same or similar binding sites reoccur in diverse protein complexes and assess their conservation within the cluster. Clusters that have

more than one non-redundant protein (at a sequence identity threshold of 90%) in the cluster are called “conserved binding site” clusters. Those clusters with only one non-redundant protein complex are considered “singletons” and usually correspond to either lineage specific binding modes or cases lacking enough conservation evidence.

Second, since the larger interfaces are more likely to be biologically relevant, the ranking score also includes a term corresponding to the number of interfacial contacts averaged over all homologous complexes (*Zcontact*). Another term in the ranking score accounts for the relevance of a given binding site cluster to the query. A position specific score matrix (PSSM) is constructed based on the binding site alignment using the implicit pseudo-count method. The aligned binding site region of the query protein is scored against the PSSM, and a sequence-PSSM score is calculated (*ZPSSM*). A higher sequence-PSSM score implies a higher probability of this site being biologically relevant for annotating the given query. In addition, we calculate the average sequence identity between the query and all cluster members over the whole structure-structure alignment (not just binding sites) to estimate the evolutionary distance between the query protein and the group of homologous structure neighbors (*Zpcnt*).

All components of the ranking score (*i.e.* PSSM, conservation, contact number, and percent identity of the alignment) are converted to Z-scores, and their weighted combination is used where weights were determined empirically.

*-* ***Validation of interactions using the PISA algorithm***

Interfaces present in PDB asymmetric units (ASU) are validated using the PISA (Protein Interfaces, Surfaces, and Assemblies server) algorithm (Krissinel & Henrick, 2007) which is considered to be one of the best methods for identifying biologically relevant interfaces present in crystal structures. PISA is an automated method for detecting macromolecular assemblies based on the analysis of interfaces and stability of assemblies reported in crystal structures. PISA uses chemical thermodynamics calculations to compute a set of macromolecular assemblies, which are expected to be stable in solution and presumed to represent the biological form of a protein in the cell.

*-* ***Assessing the accuracy of the method***

The accuracy of the IBIS method for predicting protein interaction partners and binding site locations, was found to have 88% sensitivity and a recall value of 71% (Tyagi et al, 2012). We address the question of predicting the biological interfaces that are not present in the PDB asymmetric unit and need to be reconstructed by applying crystallographic symmetry operations. We show that almost half of such interfaces can be reconstructed by IBIS without the prior knowledge of crystal parameters of the query protein.

**References**

Adzhubei IA, Schmidt S, Peshkin L, Ramensky VE, Gerasimova A, Bork P, Kondrashov AS, Sunyaev SR: A method and server for predicting damaging missense mutations. *Nat Methods* 2010, **7**(4):248-249.

Jefferson ER, Walsh TP, Barton GJ (2006) Biological units and their effect upon the properties and prediction of protein-protein interactions. *J Mol Biol* **364:** 1118-1129.

Krissinel E, Henrick K (2007) Inference of macromolecular assemblies from crystalline state. *J Mol Biol* **372:** 774-797.

Slonim N, Atwal GS, Tkacik G, Bialek W (2005) Information-based clustering. *Proc Natl Acad Sci U S A* **102:** 18297-18302.

Thangudu RR, Tyagi M, Shoemaker BA, Bryant SH, Panchenko AR, Madej T (2010) Knowledge-based annotation of small molecule binding sites in proteins. *BMC Bioinformatics* **11:** 365.

Tyagi M, Thangudu RR, Zhang D, Bryant SH, Madej T, Panchenko AR (2012) Homology inference of protein-protein interactions via conserved binding sites *PloS One,* 7(1):e28896.

Xu Q, Canutescu AA, Wang G, Shapovalov M, Obradovic Z, Dunbrack RL, Jr. (2008) Statistical analysis of interface similarity in crystals of homologous proteins. *J Mol Biol* **381:** 487-507.
